# Absent Foveal Pit, Also Known as Fovea Plana, in a Child without Associated Ocular or Systemic Findings

**DOI:** 10.1155/2018/2146826

**Published:** 2018-07-26

**Authors:** Laura Hernandez-Moreno, Natacha Moreno Perdomo, Tomas S. Aleman, Karthikeyan Baskaran, Antonio Filipe Macedo

**Affiliations:** ^1^Low Vision and Visual Rehabilitation Lab, Department and Center of Physics—Optometry and Vision Science, University of Minho Braga, Braga, Portugal; ^2^Hospital Santa Maria Maior E.P.E-Barcelos, Ophthalmology Service, Barcelos, Portugal; ^3^Scheie Eye Institute, Department of Ophthalmology, University of Pennsylvania, Philadelphia, PA, USA; ^4^Department of Medicine and Optometry, Linnaeus University, Kalmar, Sweden

## Abstract

The purpose of this report is to describe a case of bilateral foveal hypoplasia in the absence of other ophthalmological or systemic manifestations. We characterize the case of a 9-year-old Caucasian male who underwent full ophthalmologic examination, including functional measures of vision and structural measurements of the eye. Best corrected visual acuity was 0.50 logMAR in the right eye and 0.40 logMAR in the left eye. Ophthalmoscopy revealed a lack of foveal reflex that was further investigated. Optical coherence tomography (OCT) confirmed the absence of foveal depression (pit). OCT images demonstrated the abnormal structure of retina in a region in which we expected a fovea; these findings were decisive to determine the cause of reduced acuity in the child.

## 1. Introduction

The absence or poor definition of the foveal pit at the centre of the macula is normally called foveal hypoplasia [1, 2]. Marmor suggested the use of* fovea plana *instead of foveal hypoplasia when foveal cone specialization is preserved both anatomically and functionally despite the absence of a foveal pit [3]. The absence of foveal pit is commonly associated with other ophthalmic disorders such as ocular albinism, aniridia, microphthalmos, achromatopsia, and retinopathy of prematurity [2, 4, 5]. The absence of foveal pit may be accompanied with poor visual acuity and nystagmus [6, 7]. Noval and colleagues studied the macula of 286 children (mean age = 8.6 years) with optical coherence tomography (OCT) and found 9 with fovea plana. Of note, the fundus of all the 286 children involved in the study was considered normal at the time of recruitment [8, 9].

Detection and characterization of the foveal pit in children may be difficult, especially when associated with nystagmus. The use of OCT helps with such characterization but obtaining sharp images can be a challenge if the fixation is poor. With OCT features such as the extrusion of plexiform layers at the foveal centre, the morphology of the foveal pit, the length of the central outer segment (OS), and the thickness of the outer nuclear layer (ONL) can be quantitatively ascertained [1, 7]. Here we report a case of absent foveal pit without associated ocular or systemic conditions and we discuss the implications of this case for clinical practice.

## 2. Case Report

**Figure 1:**
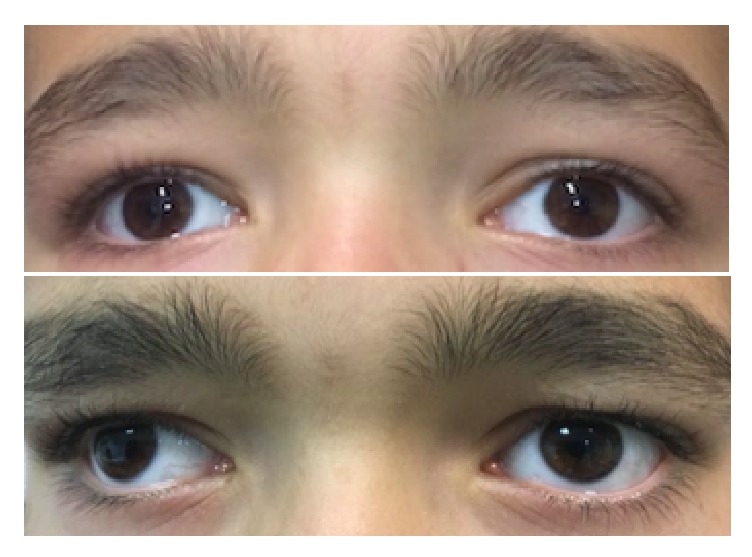
Two pictures of the external part of the eyes of our case. The physical exam excluded albinism. The top image shows near normal position of the corneal reflexes captured with a camera-flash with relatively good alignment. With the ophthalmoscope, the reflexes showed generally good alignment and often exotropia was not observable when wearing glasses. However, without glasses, as seen in the bottom image, a large angle exotropia becomes visible.

This report complies with all local laws and institutional review boards and with the principles of the Declaration of Helsinki. Informed written consent was obtained from parents. The patient was a healthy Caucasian male, aged 9 years. There was no history of amblyopia, strabismus or other eye disorders in the family and there was no parental consanguinity. As shown in Figure 1, external physical examination did not reveal obvious signs of cutaneous or ocular albinism. Parents reported that glasses were first prescribed at age 4 due to “poor vision”. Cycloplegic refraction (Cicloplegicedol 10 mg/ml) in the right eye (RE) was plano/-2.50 × 10° and in the left eye (LE) plano/-3.50 × 180°.

### 2.1. Structural Measures

**Figure 2:**
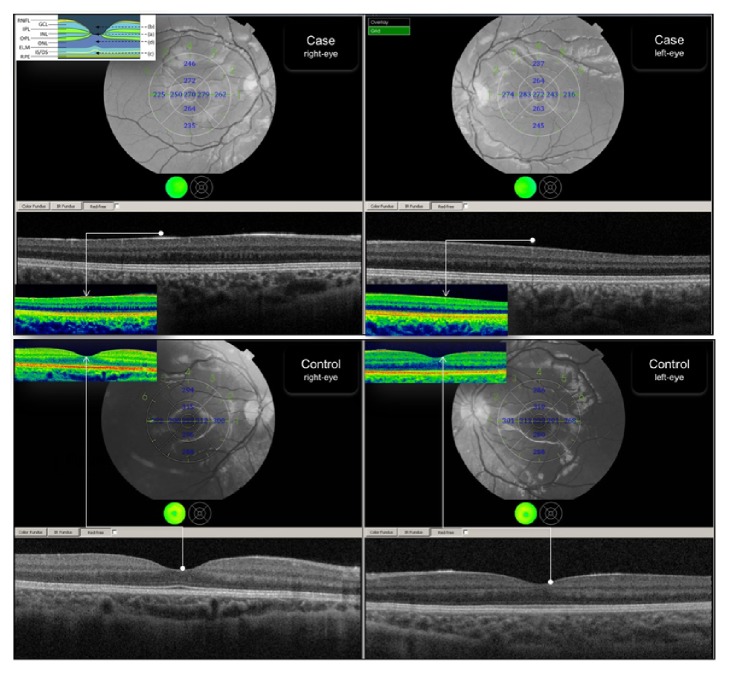
Images of both eyes of the case (top) and a control subject (bottom) obtained with spectral domain optical coherence tomography (OCT). The grayscale images are complemented with a colour-coded slice of the foveal pit (control) or the presumed anatomical location of the fovea (case). The horizontal cross-section corresponds to a horizontal section of the retina that is marked in the red-free fundus image with number 1; the OCT section shows absence of normal foveal pit in the case. All inner retinal layers are seeing crossing the anatomic foveal centre. The images of the case (top images) show a slightly thicker photoreceptor outer nuclear layer at the centre, surrounded by a ganglion cell layer that is thicker than at more peripheral locations (central ring of the retinal image), confirming the centration of this scan on the location of the anatomical fovea. Outer photoreceptor structures, such as the inner-outer segment layer and the interdigitation between the photoreceptor outer segments and the apical retinal pigment epithelium, have a normal appearance. Maps of total retinal thickness (overlapped with retinal image) show a nasal-to-temporal gradient of retinal thickness but the relative thicker parafoveal region is missing (also visible when compared with the control subject, bottom images). Of notice, the fixation position in right eye (the central ring) seems to be shifted, below the level of the optic disc. Whilst this observation might be an effect of the picture, it might also be another functional sign of an “immature” fovea and a possible explanation to more reduced vision in the right eye than the left eye.* The image at the top-left corner can be used as a “legend” to analyse the OCT findings. In that legend, (a) extrusion of plexiform layers; (b) foveal pit; (c) out-segment (OS) lengthening; and (d) out nuclear layer (ONL) widening; this legend has been adopted from [7].*

Slit-lamp examination of the anterior segment was unremarkable, but the fundus examination revealed a blunt foveal reflex. OCT images shown in Figure 2 (patient's images labeled as “case”), obtained with a Topcon 3D OCT-2000, confirmed the absence of the foveal pit at the expected location of the anatomical fovea. There was no lateral displacement of the inner retinal layers, particularly of the inner nuclear (INL) and ganglion cell (GCL) layers, which appeared as a continuous band crossing the anatomical fovea. Of interest, there was a variation of thickness of the GCL and the ONL with a gradual increase in thickness from nasal-to-temporal macula and greatest thickness near the location of the anatomical fovea. Our patient showed a significantly thicker macula when compared with an age and gender matched control (Figure 2). In the central ring the retinal thickness of our case was 270 *μ*m and 272 *μ*m for the right and left eye, respectively. The thickness of the control was 227 *μ*m in the right eye and 239 *μ*m in the left. Of note, the overall thickness of the retina in all other rings and sectors seems to be reduced when compared with the control. Biometry, performed with a Sonomed PacScan 300A biometer, revealed and axial length in our case of 23.5 mm in both eyes.

**Figure 3:**
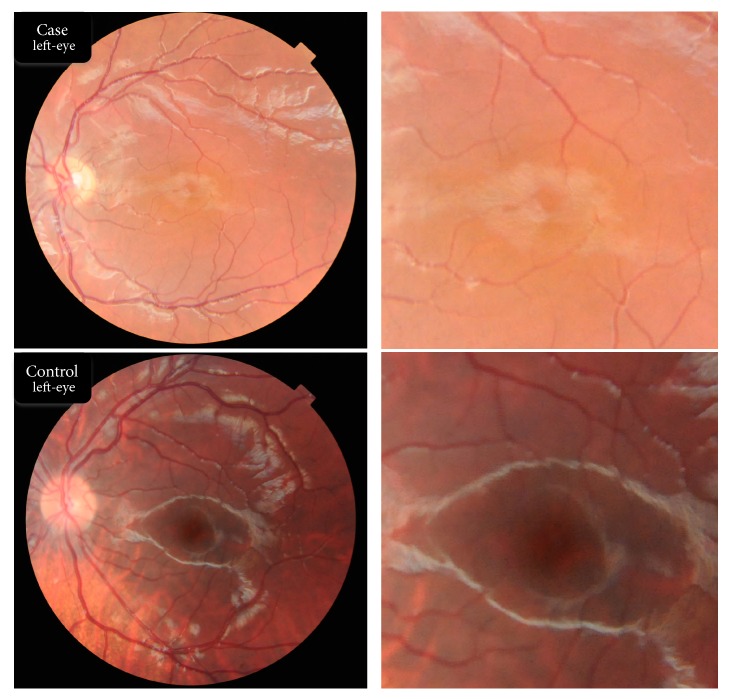
Picture of the left eye of the case (top) and the control (bottom) obtained with a fundus camera. Both foveal regions have been magnified and are shown at the right side of each retinal picture. The top image shows the absence of the foveal avascular zone in the macular area.

**Figure 4:**
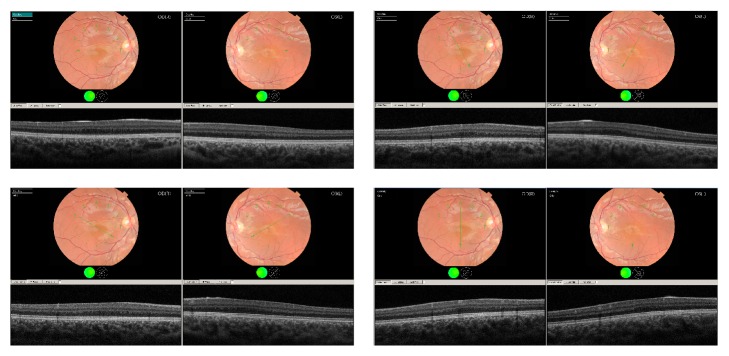
Multiple raster cuts through the suspected foveal centre of both retinas.

Fundus photographs, taken with a Topcon TRC 50DX fundus camera, are shown in Figure 3. The patient's images (Figure 3, top panel) show a poorly defined foveal zone in the left eye as well as vascular branches crossing the location of the anatomic fovea. Only one retina of the case is shown to allow the addition of the control retina (bottom panel of the figure) for comparison nonetheless both eyes of our case have similar morphological properties in images obtained from fundus camera. Four multiple raster cuts through the anatomical position of the foveal centre clearly show evidence of fovea plana (Figure 4).

### 2.2. Functional Measures

Best corrected visual acuity was 0.50 logMAR in the RE and 0.40 logMAR in the LE. Colour vision was assessed with Ishihara isochromatic plates and with the Farnsworth Munsell 100-Hue tests. The patient identified correctly 14 out of 15 Ishihara plates with the RE and 15 out of 15 with the LE. The Farnsworth Munsell 100-Hue showed a total error score of 127 for the RE and 106 for the LE, classified as “probably normal” colour discrimination for both eyes, although these error scores are expected in less than 20% of the population [10]. Cover-test at near showed an intermittent exotropia that is shown in Figure 1; horizontal nystagmus was manifest only at extreme positions of gaze, likely representing end-gaze physiologic saccades [11]. A good evidence that nystagmus was not present in primary position of gaze is the quality of the OCT and fundus images acquired.

## 3. Discussion

We used OCT to characterize the retinal structure of a young patient with an absence of the foveal pit without other ocular or systemic conditions. A normal axial length, provided by biometry, excluded microphthalmos [12]; nystagmus and iris transillumination were absent which eliminates the probability of ocular albinism [13]. This unusual anatomical configuration of the macular region has been termed by others as fovea plana [1–3, 9]. Vascularization of the, expected, foveal avascular zone, and the increased thickness of centre of the macula observed with OCT support the diagnosis. Noval and colleagues found a mean difference in retinal thickness of 43 *μ*m between the* fovea plana* group and normal fovea group [9]. In our case we found a difference of 38 *μ*m between control and case, a value that is in line with the study of Noval.

Our measures give evidence that foveal cone specialization is preserved anatomically and functionally; therefore, our case could be defined as* fovea plana*. However, the term foveal hypoplasia has also been used to describe cases with expected acuity of 0.44 logMAR, absent extrusion of plexiform layers, absence of foveal pit, presence of outer segment lengthening, and presence of ONL widening [7]. Therefore, foveal hypoplasia is likely to be the best term for our case. According to the classification proposed by Thomas and colleagues in 2011, the structural changes are consistent with a grade 4 hypoplasia. In these cases, four structural features are absent: (a) extrusion of plexiform layers; (b) foveal pit; (c) outer segment lengthening; and (d) outer nuclear layer widening [7]. However, the relative preserved visual acuity in our case despite of the markedly abnormal fovea suggests that our case can be classified as grade 2. Difficulties to match the structural features with the functional findings such as acuity have been discussed by others [5, 13]. Like us, other authors also found it difficult to perfectly match all the classification criteria. However, in our case, we speculate that astigmatism may have caused reduced retinal image quality from birth (blurred vision) that, consequently, lead to astigmatism-related amblyopia.

Our patient resembles previously reported cases where visual acuity was reduced [3]. We consider that reduced acuity is a result of an incomplete foveal development leading to defective functional development. Blurred vision caused by astigmatism in our case may have contributed to the subnormal vision. According to a recent study, astigmatism is frequent amongst children with fovea plana [5] and it is known that astigmatism above 1.50D can cause amblyopia [14, 15]. This association needs further investigation but should be taken into consideration when examining children.

We acknowledge that investigation using electrophysiology and/or genetic testing would provide further insight into this case. However, as others have shown, OCT remains the best tool to investigate foveal hypoplasia [4], and the parents of this patient did not authorize further investigation. Foveal hypoplasia has been detected at different ages following patients complaints, perhaps indicating worsening of the functional abnormalities with age in part due to associated abnormalities such as ocular/oculocutaneous albinism [1, 3, 4, 7]. Given the absence of any other abnormality, our case is unlikely to be progressive and the immature anatomy remains the most probable cause of reduced vision [7].

This diagnosis should be considered in patients with reduced visual acuity and a relatively normal fundus when seen through ophthalmoscope [8, 9]. Retinal imaging with OCT has a crucial role in the differential diagnosis in similar cases in the paediatric population.
